# Criminal behavior in alcohol-related dementia and Wernicke–Korsakoff syndrome: a Nationwide Register Study

**DOI:** 10.1007/s00406-024-01804-0

**Published:** 2024-04-13

**Authors:** Anniina Palm, Tiina Talaslahti, Risto Vataja, Milena Ginters, Hannu Kautiainen, Henrik Elonheimo, Jaana Suvisaari, Nina Lindberg, Hannu Koponen

**Affiliations:** 1 Department of Psychiatry, Helsinki University Hospital, P.O. Box 590, FI-00029 Helsinki, Finland; 2 University of Helsinki, Helsinki, Finland; 3 Primary Health Care Unit, Kuopio University Hospital, Kuopio, Finland; 4 Folkhälsan Research Center, Helsinki, Finland; 5 Finnish Institute for Health and Welfare, Helsinki, Finland; 6 Mental Health Unit, Finnish Institute for Health and Welfare, Helsinki, Finland

**Keywords:** Alcoholic Korsakoff syndrome, Alcohol-related disorders, Dementia, Crime, Mortality

## Abstract

**Background:**

Chronic heavy alcohol use may lead to permanent brain damage, cognitive impairment, and dementia. While the link between alcohol use and crime is strong, virtually no research exists on the criminal behavior of patients with the alcohol-related neurocognitive disorders of Wernicke-Korsakoff syndrome (WKS) and alcohol-related dementia (ARD).

**Methods:**

The study population included all persons diagnosed with WKS (n = 1149) or ARD (n = 2432) in Finland in 1998–2015. Data on diagnoses, mortality, and crime were obtained from Finnish nationwide registers. Crime incidences were calculated 4 years before and after diagnosis. Crime types, incidences, and mortality were compared between disorders and with the general population.

**Results:**

Altogether 35.6% of WKS patients and 23.6% of ARD patients had committed crimes in the 4 years preceding diagnosis, most commonly property and traffic crimes, followed by violent crimes. The incidence of criminal behavior decreased significantly after diagnosis; in WKS patients, the standardized criminality ratio (SCR), the ratio of observed to expected number of crimes (95% CI), was 3.91 (3.72–4.10) in 4 years before and 2.80 (2.61–3.00) in 4 years after diagnosis. Likewise, in ARD patients, the SCRs were 2.63 (2.51–2.75) before and 0.84 (0.75–0.92) after diagnosis. No significant difference emerged in mortality between persons with and without a criminal history.

**Conclusions:**

Persons with alcohol-related neurocognitive disorders frequently engage in criminal behavior prior to diagnosis, especially multiple offending. In the 4 years before and after diagnosis, crime rates declined in a linear fashion, with a marked reduction after diagnosis.

## Introduction

Criminal behavior is known to peak in people in their 20s and to decline later in life [1]. However, arrest rates in older age groups are increasing [1], and the number and proportion of older prisoners have escalated globally over the past decades [2, 3].

Older offenders differ from younger criminals in several aspects. Older offenders are more likely to be diagnosed with dementia [4, 5], physical illnesses [5, 6], affective disorders [7], and affective psychoses [4] and less likely to have personality disorders [4, 5], drug dependence [5, 7], or schizophrenia [4] than younger criminal offenders. In a recent meta-analysis, the pooled prevalence of dementia in older offenders was estimated to be 6.9%, with high heterogeneity between studies [8]. Older adults with a criminal background, especially those with severe criminality, were at higher risk of both dementia and mild cognitive impairment [9].

Previous research has shown that persons with dementia may commit crimes that are manifestations of neuropsychiatric symptoms or disinhibited behavior linked to the underlying neurocognitive disorder [10, 11]. Criminal behavior associated with dementia varies with the type of disorder, with high rates of criminal behavior seen particularly in frontotemporal dementia, both before and after diagnosis [11, 12], primary progressive aphasia, and Huntington disease [11].

The link between alcohol use and crime is strong [13, 14]. As with younger criminals, alcohol abuse is very common among older offenders [2, 5, 15]. Of older prison inmates, up to two-thirds had problems with alcohol abuse or dependence, although the actual prevalence varied in different prison populations [2]. In older forensic evaluees, alcohol abuse or dependence was also strongly associated with a diagnosis of dementia [16].

It is well established that chronic heavy alcohol use may lead to permanent brain damage, cognitive impairment, and dementia [17, 18]. In current diagnostic criteria, alcohol-related neurocognitive impairment focuses on two main syndromes: Wernicke–Korsakoff syndrome (WKS) and alcohol-related dementia (ARD) [19]. WKS is a disorder caused by thiamine (vitamin B1) deficiency, with an initial acute phase called Wernicke’s encephalopathy, followed by a chronic neuropsychiatric condition known as Korsakoff’s syndrome. Over 90% of WKS cases are linked to alcohol abuse [20]. While WKS is a discrete neurological disorder, ARD is considered a more heterogeneous dementia syndrome related to chronic heavy alcohol use [19]. Provisional diagnostic criteria by Oslin et al. [21] suggest that 35 standard drinks a week in men (28 in women) for five years is sufficient to develop ARD, although these criteria have not been independently validated and do not stem from empirical research findings.

Very little data exist on the criminal behavior of persons with alcohol-related neurocognitive disorders. Earlier knowledge on ARD and crime is entirely based on a few small studies. In their case series from a national forensic hospital in the Republic of Korea, Kim et al. [22] identified seven dementia patients, four of whom had ARD. These four ARD patients were men aged between 54 and 62 years who were incarcerated for violent crimes (assault, attempted murder, or murder). Similarly, three cases of alcohol- or substance-induced dementia and another two cases of dementia due to head trauma and alcohol were identified in Swedish forensic psychiatric examinations [23]. The subjects had committed mostly violent crimes, although the study did not specify crimes by dementia type. We were unable to find any research on the relationship between WKS and crime.

To our knowledge, earlier research on the criminal behavior of patients with alcohol-related neurocognitive disorders is almost non-existent. To address this shortfall, we designed this nationwide register-based study to explore the incidence and types of crimes committed by persons with a diagnosis of ARD or alcoholic WKS. We also examined the link between criminal behavior and mortality in these patients.

## Methods

### Data on diagnoses and mortality

In this observational study, data on persons with WKS or ARD diagnoses were obtained retrospectively from the Finnish Hospital Discharge Register (FHDR, currently named the Care Register for Health Care) [24]. The FHDR is a nationwide register launched in 1969, with mandatory data collection based on Finnish legislation. Register data contain basic patient information (date of birth, sex, municipality of residence), main diagnoses and subdiagnoses of inpatient or outpatient contacts, hospital admission and discharge dates, and information on procedures and interventions. Inpatient data are accumulated from all public hospitals, municipal health centre wards, and other (private, military, and prison) hospitals. Outpatient data are collected from all public specialized health care sectors. Since 2011, the FHDR has also collected data on all outpatient visits to public primary care. FHDR diagnoses have been recorded using the ICD-10 system since 1996. The coverage of FHDR is high, reportedly over 95% in recent years [24, 25]. Data on ca. 1.7 million persons are reported to the FHDR every year [26]. Data on population and mortality were obtained from Statistics Finland, which registers official statistics on the population and deaths (including causes of death) of all persons permanently domiciled in Finland. Data linkage was accomplished with the personal identification code issued to all persons registered in Finland. The data collection period was from 1998 to 2015 or until death. Data on mortality from death certificates were collected until the end of 2018.

### Data on crime

We collected data on criminal activities within our study population from Finnish National Police Register, a nationwide electronic database managed by the Finnish Police Administration. This comprehensive database records details of all police interactions in the country, encompassing filed crime reports, investigations, and various legal actions such as arrests, imprisonments, and warrants. The register covers all instances where the police have been alerted to a potential offense, thus including all suspected cases of crime and the dates when suspected crimes were committed [27]. We were granted access to the register by the National Police Board. In this study, crime was defined as any offense against Finnish legislation.

### Study population

The study population contained all patients (n = 3581) who had received a diagnosis of WKS (n = 1149; 841 men and 308 women) or ARD (n = 2432; 1892 men and 540 women) between 1998 and 2015 in Finland and who were aged ≥ 40 years at diagnosis. The lower age limit was set because alcohol-related neurocognitive disorders typically emerge from middle age onwards, as their development mostly involves long-term alcohol misuse. The classification of diagnoses was based on the 10th revision of the World Health Organization International Classification of Diseases (ICD-10) [28]. The study sample consisted of all cases with ICD-10 diagnoses F10.6 (alcohol-induced amnesic syndrome, Korsakoff syndrome) for WKS and F10.73 (alcohol-related dementia, alcoholic dementia) for ARD.

### Data analysis

All analyses of results were stratified according to diagnosis (WKS and ARD). Descriptive statistics were presented as means with standard deviations (SDs) or as counts with percentages. Group differences between history of criminal activity (yes, no) in the 4 years before diagnosis were evaluated using unpaired Student’s t-test and Chi-squared test. Crude cumulative rate of groups was estimated using the Kaplan–Meier method and compared between groups with the Log-Rank test. Crude and standardized estimates of crime incidence or incidence rate ratios (IRRs) were calculated using fixed-effects Poisson regression models or random-effects negative binomial regression models (unstructured correlation structure), as appropriate. The assumptions of overdispersion in the Poisson models were tested using Lagrange multiplier test. Adjusted Kaplan–Meier cumulative mortality rates were estimated using inverse probability weighting; 95% confidence intervals (CIs) were obtained by bias-corrected bootstrapping (5000 replications). Cox proportional hazard regression was used to estimate the hazard ratios (HRs) and their 95% CIs. Models were adjusted for age, sex, and year at diagnosis, when appropriate. Standardized criminality ratio (SCR) (number of actual crimes per number of expected crimes) was calculated using a subject-years methods, assuming a Poisson distribution. The numbers of observed cases and person-years at risk were counted in 5-year age groups and separately for both genders and yearly. Probabilities of crimes in an age-, gender- and event year-matched sample of the general population were calculated from data of the Finnish Police Register. The ratio of observed to expected number of deaths, the standardized mortality ratio (SMR) for all-cause deaths, was calculated using subject-years methods with 95% CIs. The expected number of deaths was calculated on the basis of sex-, age-, and calendar period-specific mortality rates in the Finnish population (Official Statistics of Finland). The statistical significance of the product terms was evaluated using a Wald test. Stata 18.0 (StataCorp LP, College Station, TX, USA) was used for statistical analyses.

## Results

In the study population, 35.6% of WKS patients and 23.6% of ARD patients had committed one or more crimes in the 4 years preceding diagnosis. Persons with WKS or ARD who had committed crimes before diagnosis were younger and more likely to be men than their peers without a criminal history. Multiple criminality was common: of those who had engaged in criminal activity before diagnosis, 61.9% of WKS patients and 54.8% of ARD patients had committed two or more crimes (Table 1).

**Table 1:** Characteristics of the study population, stratified by diagnosis and criminal behavior before diagnosis

Characteristics of the study population	Criminal behavior before diagnosis		*P*-value
	No	Yes	
WKS			
Number of persons	740	409	
Men, *n* (%)	500 (68)	341 (83)	< 0.001^c^
Crimes, *n*			
1		156	
2		100	
3 +		153	
Mean age in years at diagnosis, mean (SD)	58 (9)	54 (8)	< 0.001^d^
Person-years followed up^a^	5597	3194	
Number of deaths	429	216	
Crude mortality^b^, % (95% CI)	76.2 (66.2–85.0)	79.8 (74.7–84.5)	0.52^e^
Median survival time, years (95% CI)	10.3 (9.3–11.3)	11.0 (9.0–12.5)	
Standardized mortality ratio (95% CI)	5.30 (4.82–4.83)	6.59 (5.77–7.53)	0.010^f^
ARD			
Number of persons	1857	575	
Men, *n* (%)	1375 (74)	517 (90)	< 0.001^c^
Crimes, *n*			
1		260	
2		115	
3 +		200	
Mean age in years at diagnosis, mean (SD)	66 (9)	61 (9)	< 0.001^d^
Person-years followed up^a^	9352	3268	
Number of deaths	1160	331	
Crude mortality^b^, % (95% CI)	83.8 (76.9–90.0)	95.4 (85.4–99.3)	0.002^e^
Median survival time, years (95% CI)	5.7 (5.3–6.1)	6.8 (6.0–8.0)	
Standardized mortality ratio (95% CI)	5.23 (4.93–5.54)	6.20 (5.57–6.91)	0.008^f^

*ARD* alcohol-related dementia, *CI* confidence interval, *SD* standard deviation, *WKS* Wernicke–Korsakoff syndrome

^a^After diagnosis

^b^At the end of the follow-up period (Kaplan–Meier estimate)

^c^Chi-square test; ^d^Unpaired Student’s t-test

^e^Log-rank test

^f^Wald test after Poisson model

**Fig. 1:**
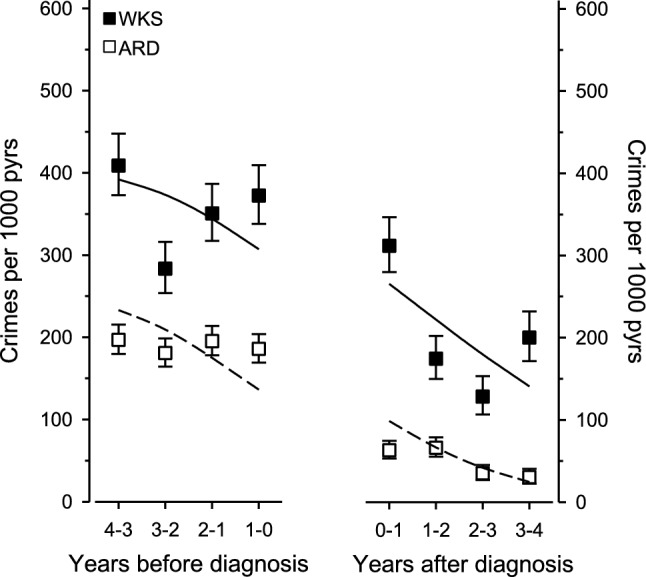
Incidence of crimes in WKS and ARD patients in the 4 years before and after diagnosis. P-values adjusted for age, sex, and calendar year at diagnosis. Error bars represent 95% confidence intervals. The lines were estimated separately using models with a quadratic term for the year before and after diagnosis. *ARD* alcohol-related dementia, *pyrs* person-years, *WKS* Wernicke–Korsakoff syndrome

In WKS patients, the incidence of crimes per 1000 person-years (95% CI) was 409 (373–448) at 4 years before diagnosis, 372 (338–410) at one year before diagnosis, 312 (280–347) at one year after diagnosis, and 200 (172–232) at 4 years after diagnosis. In ARD patients, the corresponding incidences of crimes were 197 (180–215) and 186 (169–204) at four and one year before diagnosis and 63 (53–75) and 30 (22–41) at one and 4 years after diagnosis, respectively. Criminal behavior showed statistical significance for linearity across time (adjusted for age, sex, and year at diagnosis) in both patient groups (p < 0.001) (Fig. 1).

The incidence of criminal behavior decreased significantly after diagnosis in both WKS and ARD patients (Table 2). In the WKS group, the standardized criminality ratio (SCR), i.e. the ratio of observed to expected number of crimes, was 3.91 (95% CI 3.72–4.10) in the 4 years before diagnosis and 2.80 (95% CI 2.61–3.00) in the 4 years after diagnosis. In the ARD group, the SCR was 2.63 (95% CI 2.51–2.75) in the 4 years before diagnosis and 0.84 (95% CI 0.75–0.92) in the 4 years after diagnosis.

**Table 2:** Incidence of criminal behavior in the 4 years before and after diagnosis of WKS or ARD

	Criminal behavior before diagnosis	Criminal behavior after diagnosis	IRR (95% CI)^a^
	Incidence per 1000 pyrs (95% CI)	Incidence per 1000 pyrs (95% CI)	
WKS	354 (337–372)	204 (189–220)	0.58 (0.34–0.37)
ARD	190 (181–199)	49 (43–55)	0.26 (0.18–0.20)

*ARD* alcohol-related dementia, *CI* confidence interval, *IRR* incidence rate ratio, *pyrs* person-years, *WKS* Wernicke–Korsakoff syndrome

^a^Adjusted for age, sex, and year at diagnosis

The most common types of crimes committed by WKS and ARD patients were property and traffic crimes, followed by violent crimes (Table 3). Property crimes were mainly petty larcenies; less common property crimes included damage to property, larceny, and fraud. The most frequent traffic crimes were drunken driving, traffic infractions, unlawful driving of a vehicle, and endangering traffic safety. Violent crimes were mostly assaults and illegal threats; the study population committed a total of 6 homicides and 4 negligent homicides. Alcohol crimes, or crimes against the alcohol law, signify illegally preparing, importing, or selling alcoholic beverages. The most common crimes of the “other” category were firearm offenses (meaning unlawfully possessing, manufacturing, or selling firearms or ammunition) and defamations.

**Table 3:** Number and incidence of different types of crimes in the 4 years before and after diagnosis of WKS or ARD

Type of crime	WKS		ARD		IRR (95% CI)^a^
	Number of crimes [number of persons who committed crimes]	Incidence per 1000 pyrs (95% CI)	Number of crimes [number of persons who committed crimes]	Incidence per 1000 pyrs (95% CI)	
Before diagnosis					
Traffic	680 [265]	148 (137–160)	749 [356]	77 (72–83)	**0.68 (0.60–0.76)**
Property	690 [153]	150 (139–162)	838 [209]	86 (80–92)	**0.87 (0.78–0.97)**
Violence	182 [101]	40 (34–46)	186 [107]	19 (16–22)	**0.73 (0.58–0.92)**
Alcohol	37 [31]	8 (6–11)	26 [23]	3 (2–4)	0.71 (0.41–1.24)
Sexual	9 [8]	2 (1–4)	5 [5]	1 (0–2)	0.29 (0.07–1.20)
Other	29 [26]	6 (4–9)	42 [32]	4 (3–6)	0.97 (0.53–1.77)
After diagnosis					
Traffic	282 [106]	31 (27–34)	152 [74]	12 (10–14)	**0.62 (0.50–0.77)**
Property	380 [105]	41 (37–46)	147 [62]	12 (10–14)	**0.43 (0.35–0.53)**
Violence	124 [66]	13 (11–16)	47 [30]	4 (3–5)	**0.35 (0.24–0.50)**
Alcohol	19 [17]	2 (1–3)	10 [8]	1 (0–1)	0.84 (0.36–1.92)
Sexual	1 [1]	0 (0–1)	1 [1]	0 (0–1)	.
Other	12 [11]	1 (1–2)	20 [13]	2 (1–3)	1.33 (0.60–2.96)

IRR demonstrates a statistically significant decrease, with 95% confidence interval excluding unity (in bold)

*ARD* alcohol-related dementia, *IRR* incidence rate ratio, *WKS* Wernicke–Korsakoff syndrome

^a^Adjusted for age, sex, and year at diagnosis

**Fig. 2:**
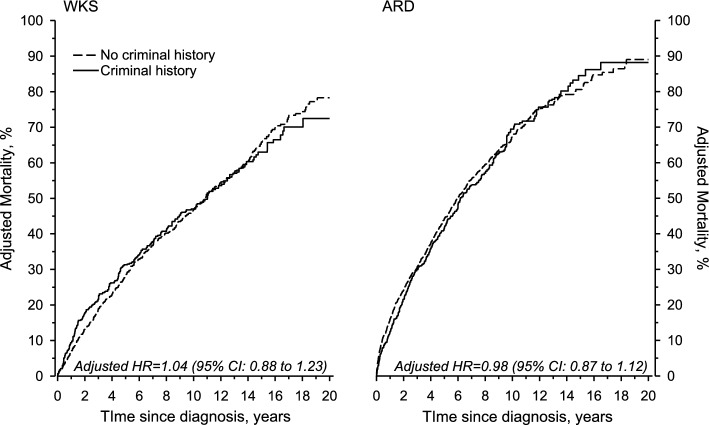
Mortality of WKS and ARD patients stratified by history of criminal activity in the four years before diagnosis. Mortality curves and hazard ratios are adjusted by age, sex, and year at diagnosis. *ARD* alcohol-related dementia, *HR* hazard ratio, *WKS* Wernicke–Korsakoff syndrome

WKS patients were significantly more likely to commit traffic, property, and violent crimes than ARD patients, both before and after diagnosis. The numbers of alcohol, sexual, and other crimes were too small for the IRR to reach statistical significance (Table 3). Regarding violent crime, there was a significant decrease in incidence post-diagnosis, with a 0.33-fold reduction observed in WKS patients and a 0.21-fold reduction in ARD patients (Table 3). No significant difference emerged in the adjusted mortality between persons with and without criminal history (Fig. 2). There was, however, a statistically significant difference in the SMRs between groups with and without criminal history before diagnosis (Table 1).

## Discussion

This is the first systematic study to examine criminal behavior in patients with alcohol-related neurocognitive disorders, and, as such, our results present an important point of reference. We found that WKS patients were 3.9 times and ARD patients 2.6 times more likely to commit crimes in the 4 years before diagnosis than the same-aged general population of the same sex. However, crime rates declined linearly in the 4 years surrounding diagnosis, with a noticeable drop after diagnosis. Multiple offending was common, with approximately 62% of WKS patients and 55% of ARD patients who had engaged in criminal activities before diagnosis having committed more than one crime, and some individuals having a record of dozens of offenses. The main types of crimes were property and traffic crimes, followed by violent crimes. Criminal behavior did not affect mortality adjusted by age, sex, and year of diagnosis.

Patients with WKS or ARD are known to have a history of significant alcohol consumption [29, 30], a high likelihood of comorbid alcohol dependence [31], and a tendency to continue to use alcohol even after diagnosis [32]. Alcohol use is strongly correlated with crime, particularly violent crimes [13] involving male offenders and male victims [33]. In Finland, 64% of male and 60% of female homicide offenders suffered from alcohol or drug abuse, and the majority committed the homicide while drunk [34]. Similarly, 72% of assault crimes were committed under the influence of alcohol when the victim was male (vs. 50% when the victim was female) [34]. Alcohol is known to elicit aggression and reduce behavioral control, probably via multiple pharmacological, cognitive, psychological, and social mechanisms [35, 36]. Alcohol has stimulative and anxiolytic properties, and it disrupts executive cognitive functions, which are central in behavioral self-regulation [36, 37]. While there has been limited research on the pre-diagnosis life circumstances of individuals with alcohol-related neurocognitive disorders, they often exhibit various socioeconomic factors that could contribute to criminal behavior, including unemployment, single marital status, and a family history of alcohol abuse [30].

Criminal behavior is particularly prevalent in neurocognitive disorders that affect the frontotemporal or frontal-subcortical circuits such as frontotemporal dementia, semantic variant of primary progressive aphasia, and Huntington’s disease [11, 12]. This is consistent with frontal lobe dysfunction being associated with aggressive and antisocial behavior. Correspondingly, alcohol neurotoxicity mainly affects the frontal lobes; persons with alcohol use disorders show decreased metabolic activity in the frontal lobes as well as frontal cortical atrophy [38].

While WKS and ARD are typically perceived as disorders affecting memory, their neuropsychological profiles also show impairment in executive control [39, 40]. The cognitive effects of alcohol may manifest as neuropsychiatric symptoms, which are highly prevalent in persons with alcohol-related neurocognitive disorders. Of institutionalized patients with WKS or other alcohol-related cognitive disorders, 96% manifested at least one neuropsychiatric symptom, most commonly irritability/lability, agitation/aggression, and disinhibition [41]. These neuropsychiatric symptoms, coupled with the typically younger age of individuals with WKS, may account for the higher likelihood of criminal behavior observed in WKS patients, both before and after diagnosis, in comparison to ARD patients.

Criminal behavior is much more common in persons with WKS or ARD than in patients with progressive dementias. In a previous study by our group, the incidence of crimes before diagnosis in men with Alzheimer’s disease was about 50 per 1000 person-years (in women, less than 10 per 1000 person-years) [12]. This rate is several-fold lower than the incidence of crimes shown in our study, which was 354 per 1000 person-years in WKS patients and 190 per 1000 person-years in ARD patients. Considering progressive dementias, even the patient group with the most criminal behavior, men with frontotemporal dementia, had a lower incidence of criminal offending than did the patients in our study [12]. Age-related factors may contribute to the higher incidence of criminal behavior observed in WKS and ARD patients, as the decline in criminal activity with age is a well-documented phenomenon. Alcohol-related neurocognitive disorders primarily affect younger age groups [42], in contrast to progressive dementias such as Alzheimer's disease.

Our results show that persons with WKS or ARD tend to commit more violent crimes than persons with progressive dementias. Violent crimes were rare in patients with Alzheimer’s disease (0.7 per 1000 person-years) or even frontotemporal dementia (4.0 per 1000 person-years) [12]. This finding is in line with previous studies; of seven dementia patients in a forensic hospital, the four patients with ARD were incarcerated for violent crimes, such as murder or assault, while the two patients with Alzheimer’s disease were incarcerated for non-violent crimes (larceny). The patients with ARD had also committed between 1 and 6 earlier crimes, whereas other dementia patients had committed none [22].

Our findings demonstrate a linear decrease in criminal activity during the 4 years surrounding the diagnosis of an alcohol-related neurocognitive disorder. Especially in ARD patients, crime rates experienced a substantial reduction following the diagnosis of an alcohol-related neurocognitive disorder. Comparing the rates before and after diagnosis, we observed a remarkable decrease to approximately one-third of pre-diagnosis levels. One possible explanation is that WKS and ARD patients may experience a progressive decline in their functional and cognitive abilities, reaching a point where they are no longer able to engage in criminal behavior. Alternatively, the act of receiving a diagnosis may signify entry into the health care system, leading to improved access to health and social care, including substance abuse treatment. Our results imply that intervention and support following diagnosis may have an impact on reducing the likelihood of engaging in criminal behavior. In Finland, a diagnosis of moderate to severe dementia or alcohol use disorder can result in revocation of driving and gun licenses, which may contribute to a decrease in traffic-related and violent offenses. Incarceration is unlikely to explain the decline in criminal activity since imprisonment sentences in Finland are reserved for serious offenses and tend to be relatively short.

Criminal behavior, particularly frequent offending, is associated with multiple psychosocial problems and premature death [27]. Even though the patients in our study had elevated mortality compared with the general population, our results did not indicate a difference in the adjusted mortality between patients with and without criminal behavior. This observation may be attributed to the fact that the elevated mortality in this patient group is primarily driven by chronic alcoholism and its related health issues, with criminality representing just one aspect of the many complex problems they confront.

### Strengths and limitations

The main strength of our study was its register-based design, which allowed us to effectively investigate patients with alcohol-related neurocognitive disorders, a population that is typically challenging to engage in clinical research. Through the utilization of Finland’s extensive nationwide electronic register databases, we successfully linked information from both health care and police registers, enhancing the comprehensiveness of our study. The high quality and reliability of Finnish health care registers, widely used in research and health monitoring and planning, further strengthened the validity of our findings. To compile a comprehensive dataset on criminal activity, we used the police register, which captures all suspected criminal offenses reported to the police, as opposed to relying solely on court data, which would only cover convictions. The use of the police register ensures the reliability of our crime-related information, due to strict legislative oversight of law enforcement and Finland’s standing as one of the least corrupt nations globally. By acquiring data on crimes directly from the police register rather than relying on self-reports, we mitigated the potential biases associated with reporting or recall. This approach provided a more objective perspective on criminal activities, although it should be acknowledged that some crimes may go unreported to the police.

The register-based design of our study, while advantageous in many aspects, also poses limitations. The health care register used here (FHDR) lacks detailed patient information such as clinical or autopsy data on individual patients. Neither were we able to obtain data on socioeconomic factors or risk factors like alcohol or tobacco use. While associations can be identified from register-based observational data, the ability to fully understand underlying mechanisms and establish causality is inherently limited in this type of study design.

Diagnoses of WKS or ARD were made during routine clinical practice, introducing the possibility of underdiagnosis and misdiagnosis. In general, dementia diagnoses recorded in Finnish health care registers are considered highly accurate [43]. However, a clear discrepancy exists between the prevalence of alcohol-related neurocognitive disorders from autopsy studies and the rates of clinically diagnosed WKS or ARD, indicating these disorders are significantly underdiagnosed [44–46]. While this could be due to the individuals being less inclined to seek medical attention, other contributing factors may include the lack of clinicians’ expertise in this field or the absence of well-established diagnostic criteria; as a result, many suspected cases might go undocumented [46].

Another limitation of our study stems from the fact that our dataset lacked information on the alcohol use disorder diagnoses of our study participants. As a result, it remains unclear whether crime rates associated with alcohol-related neurocognitive disorders are higher than those in alcohol use disorders without substantial cognitive impairment (which would imply a compounding effect of alcohol use and cognitive impairment), or if they are comparable or even lower (suggesting, perhaps, that cognitive impairment or some other factor may reduce criminal behavior). While the nationwide design of this study probably represents accurately the Finnish population, there are limitations in generalizing criminal studies to other countries or societies, as legal frameworks, justice systems, and other influencing factors vary across jurisdictions.

## Conclusion

Our study highlights that individuals with WKS or ARD show a high incidence of criminal activity in the years before diagnosis, but crime rates significantly decrease after diagnosis. The key concerns in this patient population center on the high occurrence of multiple criminal offenses and instances of violent crimes, setting them apart from the broader aging demographic. As the general population continues to age, it becomes increasingly important to recognize the unique challenges posed by older individuals with criminal histories who are also dealing with dementia. This demographic may create growing demands on police and judiciary systems as well as health care and nursing home settings [16]. To address these issues, specialized forensic services may be beneficial for older offenders [47]. Given that WKS and ARD are frequently underdiagnosed [44, 45], cognitive screening should be considered for older criminal offenders to identify these conditions. In persons diagnosed with an alcohol-related neurocognitive disorder, it is essential to assess neuropsychiatric symptoms and impairment in executive function, which may predispose to criminal behavior. These patients should also be evaluated for their driving capacity, ability to handle firearms, and financial capacity. Offering substance addiction treatment, improved health and social care, and targeted support to individuals with WKS or ARD can be valuable in preventing future criminal behavior within these patient groups.

## Data Availability

Finnish law restricts the sharing of register data. Researchers who wish to replicate our study may apply for permission from the Finnish Institute for Health and Welfare (info@thl.fi) for data from the Care Register for Health Care; from Kela (tietoaineistot@kela.fi) for data on medication purchases; and from Statistics Finland (info@stat.fi) for data on population, mortality, and causes of death. The authors are willing to provide specified portions of the statistical analysis code, such as those related to particular tests or analyses, to interested researchers upon reasonable request.
